# Sinoatrial node heterogeneity and fibroblasts increase atrial driving capability in a two-dimensional human computational model

**DOI:** 10.3389/fphys.2024.1408626

**Published:** 2024-07-30

**Authors:** Eugenio Ricci, Fazeelat Mazhar, Moreno Marzolla, Stefano Severi, Chiara Bartolucci

**Affiliations:** ^1^ Department of Electrical, Electronic, and Information Engineering “Guglielmo Marconi”, University of Bologna, Cesena, Italy; ^2^ Department of Computer Science and Engineering, University of Bologna, Cesena, Italy

**Keywords:** electrophysiology, mathematical model, sinoatrial node, pacemaking, heterogeneity, fibroblasts, high-performance computing

## Abstract

**Background:** Cardiac pacemaking remains an unsolved matter from many perspectives. Extensive experimental and computational studies have been performed to describe the sinoatrial physiology across different scales, from the molecular to clinical levels. Nevertheless, the mechanism by which a heartbeat is generated inside the sinoatrial node and propagated to the working myocardium is not fully understood at present. This work aims to provide quantitative information about this fascinating phenomenon, especially regarding the contributions of cellular heterogeneity and fibroblasts to sinoatrial node automaticity and atrial driving.

**Methods:** We developed a bidimensional computational model of the human right atrial tissue, including the sinoatrial node. State-of-the-art knowledge of the anatomical and physiological aspects was adopted during the design of the baseline tissue model. The novelty of this study is the consideration of cellular heterogeneity and fibroblasts inside the sinoatrial node for investigating the manner by which they tune the robustness of stimulus formation and conduction under different conditions (baseline, ionic current blocks, autonomic modulation, and external high-frequency pacing).

**Results:** The simulations show that both heterogeneity and fibroblasts significantly increase the safety factor for conduction by more than 10% in almost all the conditions tested and shorten the sinus node recovery time after overdrive suppression by up to 60%. In the human model, especially under challenging conditions, the fibroblasts help the heterogeneous myocytes to synchronise their rate (e.g. −82% in $\sigma_{CL}$ under 25 nM of acetylcholine administration) and capture the atrium (with 25% L-type calcium current block). However, the anatomical and gap junctional coupling aspects remain the most important model parameters that allow effective atrial excitations.

**Conclusion:** Despite the limitations to the proposed model, this work suggests a quantitative explanation to the astonishing overall heterogeneity shown by the sinoatrial node.

## 1 Introduction

The mechanisms by which the sinoatrial node (SAN) excites the atrium to start the cardiac cycle are still incompletely understood. Starting from the study by Joyner and van Capelle (1986), several computational works have investigated the driving mechanisms in animal models (mostly rabbits) (Garny et al., 2003; Oren and Clancy, 2010; Inada et al., 2014; Kharche et al., 2017; Karpaev et al., 2018; Amsaleg et al., 2022). These studies have explored the roles of gradient and mosaic configurations, presence of transitional phenotypes in the SAN periphery, and presence of specialised conduction pathways (Ricci et al., 2023).

Specific to humans, Kharche et al. (2017) used a detailed 3D anatomy combined with a simplified electrophysiological description to study the mechanisms of micro and macro re-entry onset as well as how these were modulated by the sinoatrial exit pathways (SEPs). Cellular heterogeneity was considered phenomenologically as randomly distributed SAN cells having different cycle lengths ( CL s), while fibrosis (extracellular matrix deposition) was included as an unexcitable patch at the centre of the SAN. Zyantekorov et al. (2018) investigated the effects of the SEP width and gap-junctional coupling in a 2D SAN-SEP-RA (right atrium) model. They found that narrower SEPs provided stronger conduction and that an insulating border was necessary to allow a pace-and-drive behaviour. Li et al. (2020) showed the importance of the sodium current $I_{Na}$ in determining conduction to the atrium; they showed that the SAN and SEP cells had different $I_{Na}$ , $I_{\mathrm{CaL}}$ , $I_{f}$ , and $I_{K1}$ maximal conductances. Patchy fibrosis was considered in the context of heart failure simulations and modelled as unexcitable, resistive barriers. Amsaleg et al. (2022) developed a 3D model of the SAN as well as the adjacent atrium and used it to extensively investigate the combined configurations of the gradient and mosaic models, in addition to the SEP features (number, length, and width). Sensitivity analyses were carried out on reduced models and yielded a set of parameters that achieved atrial excitation and physiological activation sequences in the full-scale 3D model. Recently, Zhao et al. (2023) proposed an anatomically detailed 2D model based on histological sections; here, the inclusion of heterogeneous properties (e.g. intrinsic rate and parasympathetic sensitivity) in the different SAN compartments (head, centre, and tail) allowed reproduction of the important experimental observations, such as shifts in the leading pacemaker location by autonomic stimulation or remodelling due to heart failure. Although the aforementioned works have provided extensive data and useful information about the atrial excitation mechanisms, none of them have considered randomised electrophysiological properties or fibroblast–myocyte interactions. Therefore, the behaviours of cellular heterogeneity and fibroblasts are still unexplored in the context of atrial driving.

Previous works by Campana et al. (2022) and Maltsev et al. (2022) have highlighted the primary role of moderated levels of heterogeneity in the SAN ionic properties that confer robustness to cardiac pacemaking. At the same time, experimental evidence of the much higher presence of fibrotic tissue in the mammalian SAN compared to the working myocardium (Camelliti et al., 2004; Csepe et al., 2015, 2016, 2017) remains to be clearly explained.

In this work, bidimensional computational models of the SAN-SEP-RA tissues featuring heterogeneity and fibroblasts were developed. State-of-the-art knowledge about the anatomy and physiology of the SAN was implemented with the aim of gaining quantitative information on the atrial driving mechanisms by the SAN as well as SAN modulation by the atrium under physiological and pathological conditions. The roles of different ionic electrophysiological properties among the SAN cells and presence of different cellular phenotypes (i.e. fibroblasts) inside the SAN were investigated.

The hypothesis proposed in this study is that both cellular heterogeneity and presence of fibroblasts in the SAN increase the robustness of driving capability in terms of the safety factor for conduction and a larger parameter space for which atrial excitation is achieved under physiological and pathological conditions. Therefore, we hypothesise that cellular heterogeneity and fibroblasts specifically contribute to overcoming hyperpolarisation from the atrium, providing a current source in the case of ionic current blocks in the SAN, and protecting the SAN from overdrive suppression under high-frequency stimulation. In the following section, we illustrate the methodology adopted to investigate these phenomena.

## 2 Methods

### 2.1 Tissue geometry and electrophysiological models

**FIGURE 1:**
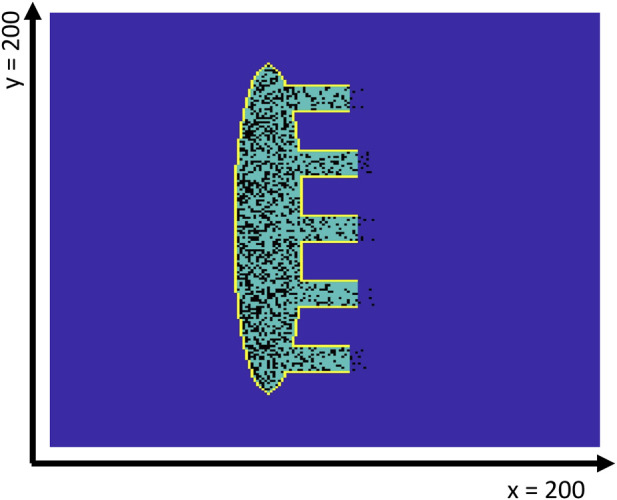
Geometry of the 2D SAN-SEP-RA human model. The yellow contour line shows the insulating border, while the dark blue interior indicates the atrial tissue. The SAN tissue is shown in green, while the fibroblasts are represented in black. Note that the latter may be present inside the atrial tissue just outside the SEPs, which are numbered #1–5 from top to bottom.

The model consists of a square matrix of 200×200 cells representing the discrete, simplified Kirchhoff network model framework (Jæger and Tveito, 2023). The sinoatrial node is located at the centre of the tissue and is modelled as an ellipse having semi-major and semi-minor axes of 75 and 12 cells, respectively (Figure 1). The SAN is surrounded by an insulating border $\left(R_{\mathrm{gap}}=\infty \text{MΩ}\right)$ that prevents interactions with the atrial tissues aside from the five SEPs; these have dimensions of 20×11 cells. Considering the Fabbri–Wilders–Severi (FWS) model cell length of 67 µm (Fabbri et al., 2017a), the above dimensions correspond to 1.34 mm, which is compatible with other experimental reports (Csepe et al., 2016) and previous computational investigations (Amsaleg et al., 2022). In addition to the FWS model, we adopted the model by Koivumäki et al. (2011) for the atrial cells (dubbed as the ‘K’ model hereafter) and the model by Morgan et al. (2016) for the fibroblasts. In addition, the behaviour of the Mazhar–Bartolucci–Severi (MBS) atrial model (Mazhar et al., 2023) recently developed by our group was tested. In this work, the latter was simplified by removing the equations describing the mechanoelectric feedback and calmodulin-kinase II activity, reducing it to 29 ordinary differential equations (ODEs) for the description of the atrial action potentials (APs).

### 2.2 Heterogeneity and fibroblasts

A linear gradient in the fibroblasts density was implemented from the left side of the SAN to the atrial tissue. In particular, the central SAN had 45.7% fibroblasts (Csepe et al., 2015, 2017; Kalyanasundaram et al., 2021; Li et al., 2023) substituting the SAN cells. The gradient allowed the SEPs to have a lower fibroblast density of 27.7 ± 16.3% vs 45.7 ± 10.9% for the central SAN, as recently reported (Li et al., 2023). The probability that a fibroblast would substitute a SAN cell depended on the gradient and on a random number between 0% and 100% extracted from a uniform distribution. If the random number exceeded the probability of not having a fibroblast, as given by the gradient (i.e. $N_{\mathrm{rand}}>$ (100% − 45.7%) in the central SAN), then the SAN cell was replaced with a fibroblast. The linear gradient dropped to zero 10 cells to the right of the SEP exit, and the fibroblasts were allowed to replace the atrial cells in these areas. However, the remaining atrial tissue was considered to be completely free of fibroblasts. With regard to cellular heterogeneity, the maximal conductances of the main ionic currents ( $P_{\mathrm{CaL}}$ , $P_{\mathrm{CaT}}$ , $g_{Kr}$ , $K_{\mathrm{NaCa}}$ , $i_{\mathrm{NaK,max}}$ , $g_{Na}$ , $g_{Ks}$ , $g_{f}$ , $g_{to}$ , and $g_{\mathrm{Kur}}$ ) along with the maximal uptake rate of the SERCA pump $\left(P_{up,basal}\right)$ of the FWS model were randomised by sampling a log-normal distribution of width 0.2. No heterogeneity was considered in the atrium. 

To improve the generalisability of these results, five different virtual SAN tissues (#1–5) were implemented for each setup (H, UF, and HF). Each of these distributions of heterogeneity, fibroblasts, or both were generated using five different random seeds for sampling the log-normal (heterogeneity) and uniform (fibroblast) distributions.

### 2.3 Gap-junctional coupling

**FIGURE 2:**
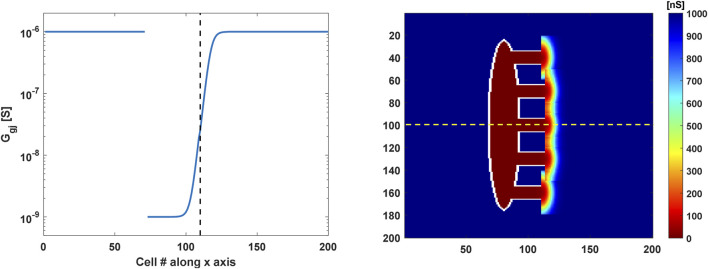
Coupling conductance profiles of the 2D SAN-SEP-RA model. (Left) Sigmoidal gradient in the gap-junctional conductance along the central SEP (yellow dashed line in the right panel). The vertical dashed black line indicates the interface between the SEP and atrium. (Right) Gap-junctional conductance values in the tissue. Bidimensional gradients of semicircular shapes were implemented in the atrium around the SEPs to allow stimulus propagation in the crista terminalis.

Coupling resistance values of 1 G Ω were each chosen for the SAN cells and fibroblasts in accordance with experimental measures and previous computational works (Verheule et al., 2001; Anumonwo et al., 1992; Rook et al., 1992; Oren and Clancy, 2010; Karpaev et al., 2018). In the atrium, $R_{\mathrm{gap}}$ was set to 1 M Ω to allow conduction velocities of 70 cm s^−1^ (Fedorov et al., 2010; Ravelli et al., 2011; Kojodjojo et al., 2006). However, we doubled $g_{Na}$ (maximal conductance of the fast sodium current) in the Koivumäki model for the whole atrial tissue to achieve conduction velocities of 100 cm s^−1^, which are typical of crista terminalis tissue (Ferrer et al., 2015; Amsaleg et al., 2022). In addition, a sigmoidal gradient of the coupling conductivity was implemented, similar to that by Amsaleg et al. (2022):

$$S=\frac{1}{R_{RA}}+\left(\frac{1}{R_{\mathrm{SAN}}}-\frac{1}{R_{RA}}\right)\frac{1}{1+e^{-\alpha x+\beta }},$$

where $R_{RA}$ and $R_{\mathrm{SAN}}$ are the respective gap-junctional resistances of the atrial and SAN tissues, α=1 , and β=37 to centre the gradients in the SEPs (Figure 2). Importantly, it was necessary to spread the gradient outside the SEPs in a semicircular shape (Amsaleg et al., 2022) to achieve driving (Figure 2). None of the parameter configurations ( $R_{\mathrm{gap}}$ in the SAN and atrium, α and β of the sigmoidal gradient) allowed the atrium to be excited if the change in conductivity occurred exclusively inside the SEPs. Additionally, the no-flux boundary conditions were applied.

### 2.4 Simulation protocols

Simulations were performed starting from single cell steady-state conditions (500 s) and lasted for 50 s. The last 5 s of the output was saved and analysed. To simulate atrial tachycardia and to investigate the responses of the SAN to overdrive suppression, a high-frequency pacing protocol was applied (Li et al., 2020). A 15 × 15 cluster of atrial cells in the top-right corner of the tissue was stimulated at 2 Hz with a current of 5 nA for 1 ms from 32 to 41.5 s before stopping the pacing. In this case, the last 10 s of the simulations output was exported to compute the sinus node recovery time (SNRT) and CL .

To assess the effects of autonomic control over the atrial driving capability and to test the SAN responses under challenging conditions, the effects of 25 nM of acetylcholine (ACh) and 1 µM isoproterenol (ISO) continuous infusion were assessed under both baseline and stimulated conditions. Moreover, 50% and 25% blocks in $I_{f}$ and $I_{\mathrm{CaL}}$ of the SAN cells were tested to investigate the robustness of atrial propagation with respect to loss of key diastolic and AP ionic currents, respectively. Finally, additional SEP widths of 7, 15, and 19 cells were investigated.

For clarity, we define ‘U’ as the uniform setup (no heterogeneity or fibroblasts), ‘H’ as the setup with SAN heterogeneity, ‘UF’ as the setup with fibroblasts and without heterogeneity, and ‘HF’ as the setup including both SAN heterogeneity and fibroblasts.

### 2.5 Analysis

The CL was computed as the time difference between two consecutive maximum points of the first derivative of the membrane voltage for the atrium or between two consecutive overshoots for the SAN, as these two methods proved to be more robust in the two cases. For the SAN, the last CL value was averaged between the cells to evaluate SAN synchronization at the end of the simulation. For the atrium, the CL values were first averaged in time and then between the cells. The safety factor (SF) for conduction was computed as the ratio of the net charge received by the neighbouring cells to the charge required to obtain a full upstroke in single-cell simulations, as in Boyle and Vigmond (2010):

$$SF=\frac{C_{m}\Delta V_{m}-Q_{\mathrm{ion}}-Q_{\mathrm{stim}}}{Q_{\mathrm{thr}}}=\frac{Q_{\mathrm{gap}}}{Q_{\mathrm{thr}}},$$

where $Q_{\mathrm{gap}}$ is computed as

$$Q_{\mathrm{gap}}=\int_{t_{a}}I_{\mathrm{gap}}\;dt.$$

Here, $I_{\mathrm{gap}}$ is the net current exchanged with the neighbours (a negative current is considered to be depolarising), and $t_{a}$ is the time interval over which $I_{\mathrm{gap}}$ is negative, as computed from the time instant at which $I_{\mathrm{gap}}$ is 1% of its negative peak to when $I_{\mathrm{gap}}$ changes sign. Because the atrial cells could be stimulated by the SAN cells very slowly, the linear dependence of $Q_{\mathrm{thr}}$ with respect to $t_{a}$ was saturated at 4 ms. Under this condition, a non-negligible charge used for depolarisation would indeed fall below the 1% of $I_{\mathrm{gap}}$ threshold, thus resulting in $Q_{\mathrm{net}}$ being smaller than $Q_{\mathrm{thr}}$ despite the cell reaching the threshold for AP firing. The SF values were computed only for the cells at the leading SEP interface, before being averaged and compared across different conditions. This was because Li et al. (2020) showed that the SAN/atrial interface provides the lowest SF . If the SAN excited the atrium across all SEPs (as in the absence of cellular heterogeneity and fibroblasts), the values were averaged between all the SEPs.

In the externally paced model tissues, the SNRT was computed as the time difference between the first atrial AP occurring after the last stimulus and the time instant of the last stimulus (Li et al., 2017, 2020). Finally, the simulated electrograms and activation time maps were generated (see the Supplementary Material for details).

Statistical differences in the CL , SF , or SNRT obtained under the different setups (H, UF, and HF, n = 5) were assessed via paired t-tests or one-sample t-tests in case of comparison with the U setup (n = 1). All data analyses were performed in MATLAB R2019b using the built-in functions, and *p*-values of 0.05 or lower were deemed significant.

### 2.6 Simulation code

The simulations were based on high-performance computing approaches. We developed an efficient CUDA/C program to update the state variables of the cells; the program implemented the forward Euler method to solve a set of ODEs by computing the current values of the state variables in parallel at each time step. Euler’s method is an instance of the bulk-synchronous parallel programming pattern (Valiant, 1990) comprising a sequence of parallel steps, where each parallel step updates the state variables. The bulk-synchronous pattern naturally maps to the architecture of a modern graphics processing unit (GPU) with a large number of computing cores that can operate in parallel. GPUs are particularly effective for implementing numerical algorithms over regular domains, such as vectors of matrices; indeed, we obtained a ∼150× speed-up with the parallel operations compared to serial implementation. 

The CUDA/C programs were written in a lightweight extension of the standard C++ programming language that included a few additional keywords and syntactic elements. A typical CUDA/C program includes ‘normal’ functions and data that are handled by the CPU as well as special functions (called kernels) that are executed by the GPU. Two different compilers were used to build the CPU and GPU executables, which were then linked in a single program.

The resulting CUDA program was implemented on a Linux server equipped with an NVidia Titan V GPU with 5,120 cores and 12 GB of device RAM, along with an AMD Ryzen Threadripper 2950x CPU operating at 3.5 GHz. A time step of 5 µs was used, and the data were undersampled at 200 µs; smaller time steps of 1 µs did not provide different results.

## 3 Results

### 3.1 Control conditions

**FIGURE 3:**
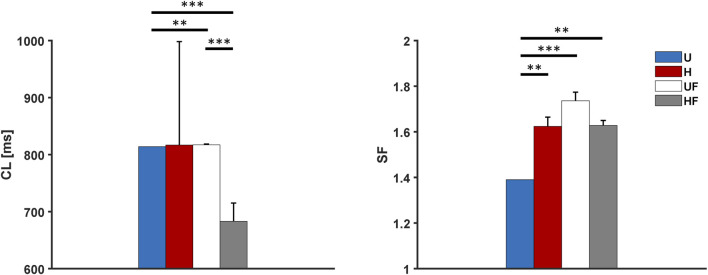
Cycle length and safety factor results for the human model under different setups: U, uniform tissue; H, SAN heterogeneity; UF, uniform SAN tissue with fibroblast; HF, SAN heterogeneity with fibroblast. *p < 0.05, **p < 0.01, and ***p < 0.001.

The behaviour of the model under the control conditions was tested first to explore the roles of heterogeneity and fibroblasts in the absence of extrinsic modifications. As shown in Figure 3, the presence of fibroblasts (UF setup) only minimally prolonged the average atrial CL compared to the uniform setup (817 ± 1 ms vs 814 ms, p < 0.01). Notably, the random fibroblast distribution showed negligible atrial $\sigma_{CL}$ (standard deviation of the CL ), while different instances of cellular heterogeneity produced widely variable CL values (817 ± 181 ms). The combination of heterogeneity and presence of fibroblasts showed a marked reduction of the CL (683 ± 32 ms) compared to those with uniform tissue and presence of fibroblasts alone (both p < 0.001); furthermore, it showed a lower CL variability than with heterogeneity alone. The large variability observed in the presence of heterogeneity (Supplementary Figure S2A) is attributed to the fact that exit blocks occur in the SEPs, with consequent alternation in the leading SEP and delay in atrial activation. The presence of fibroblasts completely abolished this phenomenon, allowing the atrial tissue to be paced at a constant steady-state rate ( $\sigma_{CL}=0$ , Supplementary Figure S2A). This can be ascribed to two main reasons. First, the fibroblasts help the myocytes to achieve better CL synchronisation (lower $\sigma_{CL}$ in the SAN) by blunting the intrinsic rate differences among the cells caused by cellular heterogeneity (Supplementary Figure S2A). Second, the fibroblasts act on the pacing and driving abilities of the SAN by increasing the SF with respect to the uniform tissue condition (1.62 ± 0.02 vs 1.45, p < 0.001). Figure 3 shows that heterogeneity alone increases the SF (1.58 ± 0.06 vs 1.45, p < 0.01) and that its combination with fibroblasts produces similar effects (1.60 ± 0.05, p < 0.01 vs U).

### 3.2 Overdrive suppression of the SAN

**FIGURE 4:**
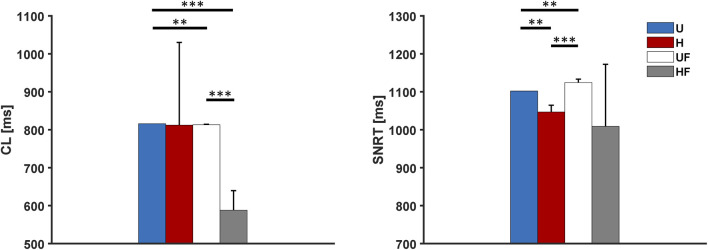
Cycle length and sinus node recovery time results for the human model after 2-Hz pacing under different setups: U, uniform tissue; H, SAN heterogeneity; UF, uniform SAN tissue with fibroblast; HF, SAN heterogeneity with fibroblast. *p < 0.05, **p < 0.01, and ***p < 0.001.

Next, we investigated the ability of the SAN to recover from external high-frequency pacing by delivering stimuli at 2 Hz to allow comparisons with prior experiments (Li et al., 2017, 2020). Excitation of the atrial tissue at this rate resulted in overdrive suppression of the SAN, with all the paced stimuli entering the SAN from all five SEPs. Once the pacing was stopped, the tissue showed CL s and $\sigma_{CL}$ similar to those under the control condition (compare the left panels of Figures 3 and 4). As in the control condition, the HF setup showed tachycardia ( CL = 588 ± 51 ms) compared to the U (815 ms), H (820 ± 218 ms), and UF (813 ± 1 ms) setups. The right panel of Figure 4 presents the SNRT values after pacing, with the heterogeneity condition showing a slightly lower value than the uniform setup (1,047 ± 18 ms vs 1,102, p < 0.01). However, in this condition, the first recovered beat originates from the SEPs, and the macro re-entry circuits with complex patterns (e.g. 2:1 exit block in the SEPs) are then stabilised in most of the tissues (Supplementary Movie S1). These observations, together with the fact that the CL s have not yet reached steady-state values after pacing, explain the high CL dispersion (Supplementary Figure S2B). Exit blocks were not observed in the UF (1,124 ± 9 ms, p < 0.01 vs U) and HF (1,007 ± 163 ms, p > 0.05 vs U) setups, but all five tissues showed re-entry activities under both cases. Thus, the fibroblasts appear to not provide faster recovery under the control conditions. Indeed, the lower average SNRT value and higher standard deviation of the HF setup are explained uniquely by the fact that tissue HF #1 had a shorter SNRT (718 ms). This occurs in all cases because the SNRT is given by the time taken by the last (*n*th) paced stimulus (at 41.5 s) to enter the SEPs, travel through the SAN, and exit through the SEPs (Supplementary Movie S1). However, this is not observed in HF tissue #1 (Supplementary Movie S2), where the (*n-1*)th stimulus excites the atrium (at 42.2 s). This is because the *n*th stimulus (at 41.5 s) collides with the (*n-2*)th stimulus (fired at 40.5 s) at the exit of SEP4. Therefore, the (*n-1*)th stimulus (fired at 41.00 s that has reached the midpoint between SEP3 and SEP4 in the meantime) does not collide with an incoming depolarisation in SEP4 and is free to excite the atria several hundreds of milliseconds before the *n*th stimulus can arrive and set the re-entry.

### 3.3 Acetylcholine administration

**FIGURE 5:**
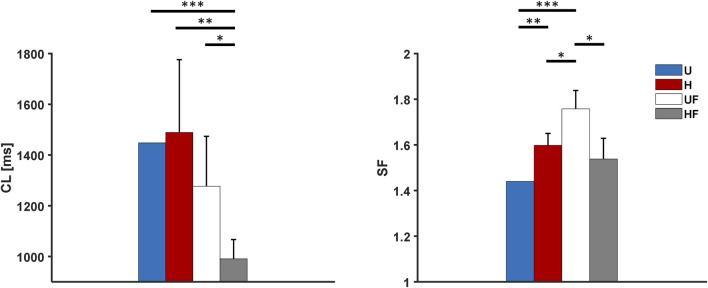
Cycle length and safety factor results for the human model with 25 nM of acetylcholine administration under different setups: U, uniform tissue; H, SAN heterogeneity; UF, uniform SAN tissue with fibroblast; HF, SAN heterogeneity with fibroblast. *p < 0.05, **p < 0.01, and ***p < 0.001.

To study the effects of parasympathetic stimulation in the model, we simulated the administration of 25 nM of ACh to the SAN. The results in Figure 5 show profound bradycardia under the uniform (1,448 ms vs 814 ms without ACh, +78% equal to the responses of single cells), heterogeneous (1,489 ± 287 ms vs 817 ± 181 ms), and fibroblast (1,277 ± 197 ms vs 817 ± 1 ms) conditions. The combination of heterogeneity and fibroblasts provides CL s closer to the physiological resting values (991 ± 76 ms, equal to an average heart rate of 61 bpm; p < 0.05 vs H and p < 0.001 vs U). However, this is still larger than the control condition (683 ± 32 ms). Moreover, both heterogeneity and fibroblasts provide strength to atrial driving in this case ( SF = 1.60 ± 0.05, 1.76 ± 0.08, and 1.54 ± 0.09 for the H, UF, and HF cases vs 1.44 for the uniform case, with p < 0.01, p < 0.001, and p > 0.05, respectively). Here, the atrial $\sigma_{CL}$ in the H setup is still large owing to the occurrence of exit blocks and leading SEP alternation (Supplementary Figure S2C).

### 3.4 Overdrive suppression of the SAN with acetylcholine administration

**FIGURE 6:**
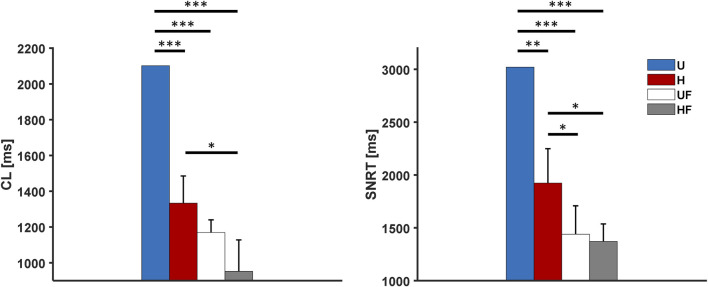
Cycle length and sinus node recovery time results for the human model with 25 nM of acetylcholine administration after 2-Hz pacing under different setups: U, uniform tissue; H, SAN heterogeneity; UF, uniform SAN tissue with fibroblast; HF, SAN heterogeneity with fibroblast. *p < 0.05, **p < 0.01, and ***p < 0.001.

We next tested a challenging condition in which the SAN underwent simultaneous 25 nM of ACh infusion and external 2 Hz pacing, as in case of the *ex vivo* human hearts in Li et al. (2017, 2020). In this setting, the CLs showed values of 2,102 ms, 1,333 ± 152 ms, 1,169 ± 71 ms, and 952 ± 176 ms for the U, H, UF, and HF cases (p < 0.001 for all setups vs U; p < 0.05 for HF vs H). The SAN was again completely suppressed by the depolarisations travelling through all the SEPs. For the SNRT , Figure 6 interestingly indicates that the H, UF, and HF cases showed strikingly fast recoveries of pacemaking, contrary to that in the control case: 1,923 ± 325 ms for the heterogeneous tissue, 1,440 ± 269 ms with the fibroblasts, and 1,206 ± 141 ms in presence of both heterogeneity and fibroblasts vs 3,020 ms for the uniform model (p < 0.01, p < 0.001, and p < 0.001, respectively). The decrease in SNRT by the addition of fibroblasts to the tissue is larger than that given by the presence the presence of cellular heterogeneity alone. Compared to the results without ACh administration, the uniform setup showed SNRT prolongation of +174% ; with this setup, the SAN depolarisations originated in the middle of the SEPs as in the case without ACh, but no re-entry was established even though 2:1 exit blocks occurred for the first two beats (Supplementary Figure S4 and Supplementary Movie S3, $\sigma_{CL}$ = 950 ms). In presence of heterogeneity, re-entry circuits were formed in all tissues, but the administration of ACh increased the number of exit blocks after pacing cessation. Given the longer CL , was larger, there were clusters of spontaneous cells that fired APs before the arrival of the re-entry stimulus, later merging with it at the entrance of the leading SEP. This occurred in the presence of fibroblasts as well, but only tissue #3 of the HF setup showed exit blocks from the SEPs. Supplementary Figure S2D indeed shows very large atrial $\sigma_{CL}$ in the H setup, with a substantially lower value for the UF setup and almost no variability in the HF case. This again suggests that fibroblasts help the SEPs in capturing the atrium (Supplementary Figure S4).

### 3.5 Isoproterenol administration

**FIGURE 7:**
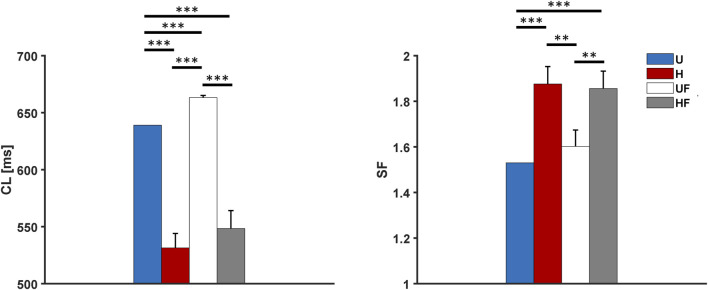
Cycle length and safety factor results for the human model with 1 µM of isoproterenol administration under different setups: U, uniform tissue; H, SAN heterogeneity; UF, uniform SAN tissue with fibroblast; HF, SAN heterogeneity with fibroblast. *p < 0.05, **p < 0.01, and ***p < 0.001.

After the experiments with ACh infusion, we evaluated the model responses to sympathetic stimulation. In this case, given the on/off behaviour of the FWS model with respect to adrenergic activation (calibrated to 1 µM release), it was not possible to use other concentrations, as reported in previous experiments (1 nM in Li et al. (2020)). We consequently simulated the infusion of 1 µM ISO, which shortened the CL in all cases with respect to the control: U = −21% (639 vs 814 ms), H = −30% (531 ± 13 ms vs 764 ± 183 ms), UF = −19% (663 ± 2 ms vs 817 ± 1 ms), and HF = −18% (548 ± 16 ms vs 665 ± 55 ms). The H setup did not show any exit block, with consequently reduced CL and CL variability (Figure 7 and Supplementary Figure S2E); this is attributed to the remarkable increase ( > 20%) in the SF in both the H and HF cases compared to the uniform one (1.88 ± 0.08 and 1.86 ± 0.08 vs 1.53, respectively; p < 0.001 in both cases). Although the UF setup showed a milder increase to 1.63 ± 0.07 (p > 0.05 vs U), it did not show any exit blocks.

### 3.6 Overdrive suppression of the SAN with isoproterenol administration

**FIGURE 8:**
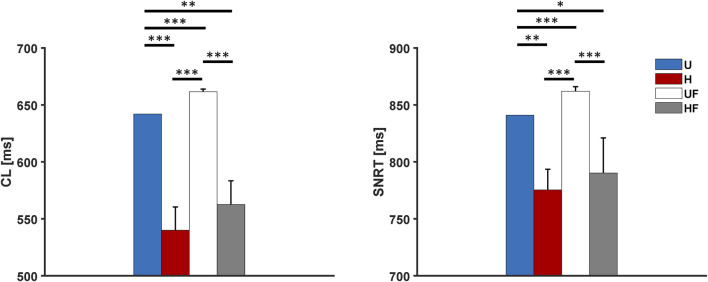
Cycle length and sinus node recovery time results for the human model with 1 µM of isoproterenol administration after 2-Hz pacing under different setups: U, uniform tissue; H, SAN heterogeneity; UF, uniform SAN tissue with fibroblast; HF, SAN heterogeneity with fibroblast. *p < 0.05, **p < 0.01, and ***p < 0.001.

We next added external 2 Hz pacing along with 1 µM ISO infusion. The models showed average CL values similar to those of the non-paced conditions (Figure 8). Despite the SNRT in the U setup being shorter than that without ISO (841 ms vs 1,102 ms, −24%), the H and HF cases showed much faster recoveries ( SNRT = 775 ± 18 ms and 790 ± 31 ms, p < 0.01 and p < 0.05 vs U, respectively) compared to their control values. The UF setup showed the largest SNRT (862 ± 4 ms, p < 0.001 vs U, H, and HF), which is still less than that without ISO.

### 3.7 Funny current block in the SAN

**FIGURE 9:**
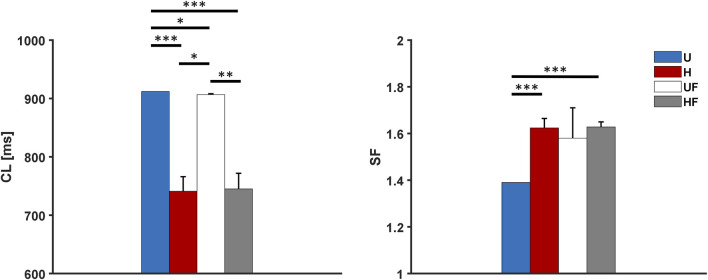
Cycle length and safety factor results for the human model with 50% funny current block under different setups: U, uniform tissue; H, SAN heterogeneity; UF, uniform SAN tissue with fibroblast; HF, SAN heterogeneity with fibroblast. *p < 0.05, **p < 0.01, and ***p < 0.001.

To evaluate if the model was robust to loss of depolarising current during diastole and if the heterogeneity and fibroblasts contributed to its strength, the funny current $I_{f}$ was blocked by 50% in the SAN (Figure 9). As a consequence, a 12% CL prolongation was observed in the uniform tissue compared to the control tissue (912 ms vs 814 ms), just as in the single-cell simulations with the FWS model (Fabbri et al., 2017a). Similar results were obtained in the HF case (average +10.7% , 745 ± 27 ms vs 665 ± 55 ms without blocking); however, a counterintuitive tendency toward CL shortening was noted in the H setup ( −9% , 741 ± 25 ms vs 817 ± 181 ms). This can be explained by the presence of exit blocks in the control H setup, which induce the longer average CL in the atrium despite the fact that the CL is longer with the $I_{f}$ block within the SAN (741 ± 25 ms vs 662 ± 26 ms), as expected.

Both setups show lower CL (741 ± 25 ms and 745 ± 27 ms vs 912 ms, with p < 0.001, p < 0.001, and p < 0.001 for H, UF, and HF, respectively) and higher SF (1.62 ± 0.04 and 1.63 ± 0.02 vs 1.39, with all p < 0.001) values compared to the uniform SAN case. With the UF setup, only three tissues achieved atrial driving; these showed CL s similar to that of the U case (907 ± 2 ms, +11% prolongation compared to control), along with similar SF s (1.58 ± 0.13, p > 0.05).

### 3.8 L-type calcium current block in the SAN

The L-type calcium current determines the slow SAN AP. Therefore, we simulated a 25% reduction in $I_{\mathrm{CaL}}$ to assess tissue robustness to a loss of the main current during the upstroke phase. This block leads to loss of driving in the U and in all H models. Intriguingly, three out of the five UF models and four out of the five HF models showed pace-and-drive behaviours. The UF tissue #1 as well as HF tissues #3 (Supplementary Movie S4) and #4 showed 2:1, 3:1, and 2:1 exit blocks, respectively, with consequent extreme atrial bradycardia ( CL = 2,209 ms or 27 bpm with only two APs in 5 s, CL = 2,583 ms or 23 bpm, and CL = 1,797 ms or 33 bpm). The HF tissue #5 also showed complex electrical activity, with re-entry excitation showing one exit block in the last 5 s of the simulation and a resulting CL of 1,142 ± 463 ms. Only HF tissue #2 showed a regular physiological rate ( CL = 806 ± 0 ms, 74 bpm), but its activation started from the centre of #SEP2 (Supplementary Figure S5). The UF tissues #2 and #4 show stable driving but at a slightly bradycardic rate ( CL = 1,073 ± 0 ms and 1,076 ± 0 ms, both equal to 56 bpm). In all these cases, the SF appeared to be slightly reduced compared to that of the HF model under other conditions (Supplementary Figure S3).

Supplementary Figure S3 summarises the SF results for the four setups under the different conditions. In the UF condition (panel C), there was frequency dependence of the SF , which increased with longer CL s (e.g. with ACh or $I_{f}$ block). In addition, SAN frequency synchronisation was worse in the HF case than the H and UF cases with both $I_{f}$ and $I_{\mathrm{CaL}}$ current blocks (Supplementary Figure S2, panels G and H). In all other cases, the combination of heterogeneity and fibroblasts produced lower or equal $\sigma_{CL}$ values within the SAN.

### 3.9 Effects of SEP width and mosaic configuration on atrial driving

To gain further understanding of the driving mechanisms, two additional model parameters were investigated, namely the width of the exit pathways and cellular organisations at their interfaces. Table 1 reports the effects of the SEP width on the driving capability of the SAN, showing that there is a lower limit (width = 11 cells) for propagation. The results are similar for both the K and MBS models, with no modulations of the CL values with respect to the SEP width. However, the conduction velocity along the leading SEP increases proportionally with the width and a corresponding decrease in the conduction time. The MBS model required a mosaic configuration in addition to the design described previously to be able to pace the atrium; this included a gradient of atrial cells interspersed in the SEPs, without which it was not possible to obtain propagation in the uniform SAN condition. When considering heterogeneity (without mosaic), only one of the five models achieved very poor driving, with a 3:1 exit block and consequent pronounced bradycardia ( CL = 1,932 ms). However, even with the mosaic (uniform) configuration, the atrium was effectively activated in only three of the five models ( CL = 814 ± 0 ms).

**TABLE 1:** Effects of SEP width on pacing and driving under the K and MBS models. P but no D, pace but no drive; CV, conduction velocity; CT, conduction time; CL, cycle length; SF, safety factor.

**SEP width** [**# of cells**]	7	11	15	19
K model				
**CL** [**ms**]	P but no D	814 ± 0	814 ± 0	814 ± 0
**SEP CV** [**cm** ${\text{s}}^{-1}$ ]	-	0.35	0.41	0.52
**SEP CT [ms]**	-	380	320	250
**SF**	-	1.45 ± 0.03	1.48 ± 0.01	1.63 ± 0.08
MBS model				
**CL [ms]**	P but no D	814 ± 0	814 ± 0	814 ± 0
**SEP CV** [**cm** ${\text{s}}^{-1}$ ]	-	0.27	0.38	0.49
**SEP CT [ms]**	-	490	350	270
**SF**	-	1.38 ± 0.15	1.45 ± 0.23	1.31 ± 0.09

### 3.10 Combining heterogeneity, fibroblasts, and neural modulation

**FIGURE 10:**
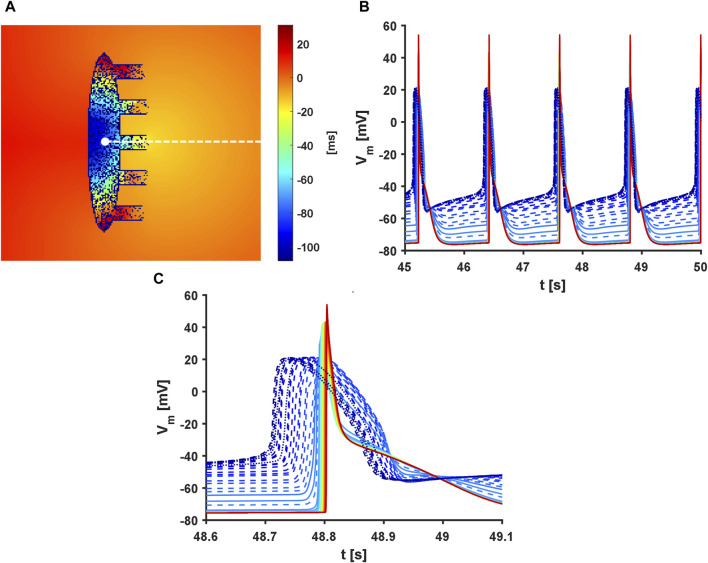
Activation sequence in the full model. Presence of heterogeneity and fibroblasts along with administration of 25 nM of acetylcholine and 1 µM of isoproterenol were considered. In addition, $R_{\mathrm{gap}}$ was set to 10 and 100 M Ω for the SAN cells and fibroblasts, respectively. **(A)** Activation time map. **(B)** Action potentials along the dashed white line of panel **(A)**. **(C)** Magnification along the *X*-axis of panel **(B)**. The colour code is consistent with activation times from panel **(A)**. The SAN cells are plotted as dashed lines, fibroblasts as dotted lines, and atrial cells as solid lines.

A final set of simulations including heterogeneity, fibroblasts (HF tissue #3 with K model), and simultaneous administration of 25 nM of ACh as well as 1 µM of ISO (hereafter called the ‘full’ model), was implemented to model a condition as close to the SAN physiology as possible. To reproduce the physiological activation sequence, $R_{\mathrm{gap}}$ was lowered to 10 and 100 M Ω for the SAN cells and fibroblasts, respectively. In addition, the mosaic model was implemented according to the MBS model simulations, while the FWS model was modified to account for the simultaneous adrenergic and cholinergic effects in an additive manner (Supplementary Material). This setting provides a CL of 1,192 ms, with the activation starting from the left central part of the SAN and exciting the atrium through SEP3 after roughly 80 ms (Figure 10 and Supplementary Movie S5). The propagation wave then re-enters the SAN from SEP1 and SEP5, where it collides with the depolarisations arriving from the head and tail of the SAN. The SF in this condition was 1.56.

When the $R_{\mathrm{gap}}$ of the fibroblasts was retained at its original value of 1 G Ω , a more physiological CL of 961 ms and higher SF of 1.75 were achieved; however, atrial excitation occurred after ∼ 120 ms through SEP#5, and the slow CV inside the SAN allowed entry of the depolarisations from the other SEPs (Supplementary Movie S6). Interestingly, pacemaking activity was completely suppressed in the absence of the fibroblasts.

## 4 Discussion

The results reported above indicate that the proposed model shows atrial driving across both physiological and pathological conditions affecting the SAN. Furthermore, the simulation results suggest that heterogeneity and fibroblasts increase the driving capability of the SAN while also improving its recovery from overdrive suppression.

### 4.1 Heterogeneity and fibroblasts increase robustness of atrial driving

The important and novel roles of cellular heterogeneity and fibroblasts displayed by the proposed model include increasing the ability of the human SAN to drive the atrium (Figures 3, 5, 7, and 9). In all conditions, the H configuration showed substantially higher SF values ( >+10 %); this is likely due to the presence of stronger cells (i.e. expressing, for example, more $I_{\mathrm{CaL}}$ ) that deliver more current at the SEP interfaces. The UF and HF setups showed similar behaviours under most conditions since the fibroblasts acted as current sources and allowed the SAN cells to provide more charge to the atrial tissue, as described below. The information in Supplementary Table S2 supports this observation by reporting how the heterogeneity and fibroblasts determine more depolarised maximum diastolic potentials ( MDP s) as well as higher upstroke velocities, especially in the leading SEP, compared to when they are absent. The contributions of cellular heterogeneity to the ionic properties have long been deemed central to robust pacemaking (Boyett et al., 2000). With regard to fibroblasts, experimental and computational studies on the ventricular tissues (Fahrenbach et al., 2007; Xie et al., 2009) have allowed hypothesising their ability to influence the source–sink relationship for effective stimulus propagation in the SAN (Csepe et al., 2015). Indeed, a possible first validation of the present results can be obtained from co-cultures of fibroblasts and ventricular myocytes (Miragoli et al., 2007): in these experiments, the percentage of myocyte preparations showing spontaneous activity was above zero only when a minimum amount of fibroblasts (20%) was present, and this percentage was maximum ( > 80%) with 50% fibroblast density. Considering that fibroblast–myocyte coupling has been demonstrated *in vivo* recently in mouse infarcted ventricular tissue (Wang et al., 2023), our simulations point toward the role of the depolarised resting potential of the fibroblasts in robust pacemaking.

The administration of 1 µM of ISO showed synergistic effects with both the H and HF configurations, producing greater increases in the SF than that for the uniform case (Supplementary Figure S3). In fact, if we exclude the SF increase in the UF setup in the presence of ACh (likely due to the myocytes undergoing a longer and hyperpolarised diastolic phase, that allows fibroblasts to supply them more charge, only ISO provided higher SF values than the other conditions (control, ACh, and current blocks) for the same configurations (Supplementary Figure S3).

The full model showed an SF value (1.56) closer to the average obtained with ACh administration (1.54 ± 0.09) than that with ISO infusion (1.86 ± 0.08). When considering a weaker coupling of the fibroblasts (original $R_{\mathrm{gap}}$ value of 1 G Ω ), this is sufficient to substantially increase the SF toward the ISO value (1.75); this is consistent with the results obtained from coupled rabbit SAN and atrial cell models, which showed a substantial dependence of the SF on $R_{\mathrm{gap}}$ (Cacciani and Zaniboni, 2015). An additional beneficial consequence of the increased $R_{\mathrm{gap}}$ in the full model is the avoidance of bradycardia (62 vs 50 bpm). Nevertheless, both effects are obtained at the cost of losing phase synchronisation inside the SAN (lower apparent conduction velocity), a fact that may facilitate re-entry onset (Supplementary Movie S6). Most interestingly, when the fibroblasts were removed from the full model, the SAN tissue lost automaticity. This supports the importance of fibroblasts in mitigating atrial hyperpolarisation, thereby protecting depolarisation of the SAN myocytes and allowing atrial driving.

### 4.2 Heterogeneity and fibroblasts provide protection from reductions of key ion currents

The simulations with $I_{f}$ and $I_{\mathrm{CaL}}$ blocks showed that heterogeneity protected the SAN from loss of key ionic currents when coupled to the atrium (Figure 9), confirming its beneficial actions commonly found in isolated pacemaker tissues (Campana et al., 2022; Maltsev et al., 2022). For fibroblasts, stronger beneficial effects were observed, allowing the SAN to pace the atrium despite a 25% $I_{\mathrm{CaL}}$ block with the UF and HF setups, while the U and H tissues remained quiescent. Similar to the increase in SF , this is attributed to the depolarisations of the MDPs and especially the increase in the $dV/dt_{\max}$ of the SAN cells in the leading SEP owing to the synergism of heterogeneity and fibroblasts (Supplementary Table S3).

### 4.3 Heterogeneity and fibroblasts improve recovery from overdrive suppression

In this work, the indirect SNRT , obtained as the time interval between the first atrial AP and last paced beat (Li et al., 2017, 2020), was used to quantify the ability of the SAN to recover its driving capability after overdrive suppression. In the heterogeneous tissue under the control conditions (no blocks or drug administration), the first spontaneous beat propagates from the middle of the SEP slightly before than it does in the uniform tissue. After this beat, re-entry is established in all five models, but they do not stabilise on specific SEPs as in the case with fibroblasts. The latter configuration does not have exit blocks, in line with the observations of Zhao et al. (2023), who observed lower exit block occurrences upon sodium current block or ACh administration compared to when the fibroblasts were not considered in remodelling owing to heart failure. This suggests that even though fibroblasts provide strength to atrial driving, they also lead to macro re-entry stabilisation in the present model.

After pacing at 2 Hz, all setups showed *SNRT* values close to experimental observations in two explanted human hearts: U = 1,102 ms, H = 1,047 ± 18 ms, UF = 1,124 ± 9 ms, and HF = 1,063 ± 163 ms vs the values of 1,020 and 1,459 ms reported by Li et al. (2020). When corrected for the basal CL , the SNRT of the uniform setup (288 ms) was in agreement with the extant computational reports (167 ms with pacing at 950 ms; Li et al. (2020)). However, after external pacing, no exit blocks were observed in the experiments of Li et al. (2020) and in the computational model reported by Zhao et al. (2023). In both these cases, the exit blocks were obtained only with reduced sodium current and ACh administration or under heart failure remodelling conditions, which also led to re-entry.

Furthermore, simulations with administration of 25 nM of ACh showed that the fibroblasts greatly reduced the SNRT compared to the heterogeneous tissues (−23% for UF and −29% for HF), which in turn allowed earlier recovery than in the uniform case (−36%, Figure 6). In particular, the SNRT values in the UF and HF setups with ACh administration were much closer to the values without ACh (1,479 ± 223 ms vs 1,124 ± 9 ms and 1,371 ± 165 ms vs 1,007 ± 163 ms) than that with the H setup (1,923 ± 325 ms vs 1,047 ± 18 ms). Our finding of SNRT = 3,020 ms with the U setup, which shows nodal dysfunction (corrected indirect SNRT> 525 ms), is in line with the *ex vivo* experimental findings on dogs (Lou et al., 2013, 2014) and humans (Li et al., 2020). Modelling work on humans (Li et al., 2020) congruently showed atrial pauses of > 5 s due to exit blocks at the SEP exits upon ACh application when the fast sodium current was blocked by 20%. In the rabbit model by Karpaev et al. (2018), the durations of the pauses were proportional to the number of fibroblasts. This discrepancy may be ascribed to the different basal heart rate. No pauses were reported in the model by Zhao et al. (2023). During pacing, none of the models showed filtering of the paced stimulus through the SEPs, as previously observed in optical mapping experiments in human hearts at frequencies lower than or equal to 2 Hz (Li et al., 2017).

### 4.4 Heart rate modulation by heterogeneity and fibroblasts

In the uniform setup, the models showed CL s equal to the single-cell values (814 ms); this is shorter than values reported in earlier computational works (vs 883 ± 1.7 ms in Amsaleg et al. (2022), 999 ms in Li et al. (2020), and 930 ms in Zhao et al. (2023)) but still within the range of human heart rates (74 vs 60−100 bpm).

The occurrences of exit blocks in the baseline H setups explain the large atrial $\sigma_{CL}$ and average values close to those of the uniform setup. Interestingly, the simultaneous presence of heterogeneity and fibroblasts resulted in lower CL and $\sigma_{CL}$ . Unlike the observations in the preliminary human single-cell simulations reported by our group (Ricci et al., 2021), when the SAN cells were hyperpolarised by the atrium, the duration for which their membrane voltages were below the resting potential of the fibroblasts (−50 mV) increased. Thus, the fibroblasts acted as current sources over a larger portion of the diastolic depolarisation ( DD ) phase, accelerating it and strengthening atrial depolarisation. In addition, owing to their depolarised resting potentials, the fibroblasts softened the intrinsic rate differences due to parameter randomisation among the cells by pushing them toward similar beating frequencies (Supplementary Figure S2) and provided enough current to avoid exit blocks from the SEPs, further supporting the hypothesis of protective actions of the fibroblasts from hyperpolarisation.

By itself, the presence of fibroblasts prolongs the CL minimally (Figure 3); this small effect is consistent with the findings of Karpaev et al. (2018) but contrasts in amplitude with the larger prolongation found by Oren and Clancy (2010), with the experiments of Csepe et al. (2015) and Fahrenbach et al. (2007), as well as with our previous results in isolated human SAN models (Ricci et al., 2021). However, this discrepancy may be explained by the voltage difference between the resting potential of the fibroblasts and MDP of the SAN cells when they are also coupled to the atrium, according to the mechanism described above.

Interestingly, when ACh is applied, the combination of heterogeneity and fibroblasts prevents the occurrence of exit blocks in the H model and shortens the CL in the U setup (1,448 ms, +78%, slightly longer than that reported by Li et al. (2020) but in line with the findings of Zhao et al. (2023) who obtained +28% prolongation with 10 nM of ACh), thereby avoiding bradycardia (991 ± 76 ms, equal to 61 bpm) and reducing CL variability (Figure 5). During ISO administration, the HF setup also showed a tendency to limit the tachycardia (109 vs 113 bpm) achieved in the H setup (Figure 7), in agreement with the lower average rates obtained with the fibroblasts alone (90 bpm). Therefore, from this perspective, the fibroblasts provide robustness to atrial driving but reduce heart rate flexibility at the same time.

In the full model, a heart rate of 50 bpm was obtained; this slight bradycardia was the consequence of the reduced $R_{\mathrm{gap}}$ of both the SAN cells and fibroblasts needed to achieve a physiological atrial activation time. Furthermore, when considering the simultaneous simulated infusion of ACh and ISO, the model shows the predominance of cholinergic over adrenergic effects, in line with the experimental observation that the intrinsic rate (e.g. in the denervated heart) is higher than the *in vivo* rate owing to the absence of the large basal parasympathetic tone (Opthof, 2000; Peters et al., 2020).

### 4.5 Heterogeneity and fibroblasts shift the leading pacemaker location

Depending on both the randomised ionic properties and the fibroblast distribution, clusters with different intrinsic properties (e.g. beating rate) are formed inside the tissue and become dominant. The activation sequence originates according to the locations of these clusters (Supplementary Figure S5 and Supplementary Movie S6) and more distally from the atrium compared to the uniform model.

Thus, the physiological levels of heterogeneity ( σ=0.2 for the log-normal distribution (Sobie, 2009; Campana et al., 2022; Maltsev et al., (2022)) and fibroblasts (Csepe et al., 2016, 2017; Li et al., 2023) can overcome the atrial sink effect to shift the leading pacemaker (LPM) location at least at the coupling values adopted. Interventions that decrease the strength of the source or increase the sink, such as reducing $R_{\mathrm{gap}}$ in the full model, administering 25 nM of ACh, or blocking $I_{f}$ , shift the LPM away from the SEP exits. Consequently, the electrical excitation follows a more regular pattern of activation, propagating from the left part of the SAN toward the atrium (Figure 10).

Interestingly, maintaining a fixed distribution of the diffused fibroblasts while changing the heterogeneity distribution always resulted in atrial activation from the same SEP (not shown), suggesting that the fibroblasts may guide propagation. This is further strengthened by symmetrical simulations in which the distribution of cellular heterogeneity was fixed and that of the fibroblasts was changed, resulting in atrial activation via different SEPs.

### 4.6 Gradients in the gap-junctional conductivity are necessary to achieve atrial driving

A steep gradient in the gap-junctional coupling was found to be necessary to achieve atrial driving. This suggests that the most important parameter for achieving robust atrial excitation is intercellular coupling, as highlighted in previous works (Kharche et al., 2017; Inada et al., 2014; Amsaleg et al., 2022). Indeed, the slope, half value, and transition shapes inside and outside the SEPs were seen to deeply affect propagation, with only a fine balance among these parameters allowing propagation in the model, as reported in the Methods.

### 4.7 Effects of exit pathway widths and numbers

The number of SEPs does not influence the average CL or the SF , suggesting that the designed coupling gradient allows the SAN to be a sufficiently strong source that is unaffected by increased sink (Fabbri et al., 2017b). With regard to the SEP widths, the simulations show that there are lower limits on the numbers of cells comprising the exit pathways in both the K (Koivumäki et al., 2011) and MBS (Mazhar et al., 2023) models for excitation of the crista terminalis, as found in the detailed 3D model by Amsaleg et al. (2022). However, Li et al. (2020) managed to achieve SAN–atrial propagation with one SEP as narrow as three cells, even in the context of simulated heart failure (20% $I_{f}$ block; $I_{Na}$ blocks in the SAN, non-conductive fibrosis in the SAN and SEP; 5% $I_{Na}$ block in the atrium). This difference could possibly be explained by the fact that the authors scaled the maximal conductance of the sodium current in the SEP by a factor of five, thereby increasing the ability of the pathway to capture the atrium.

As the SEP widths increased, slight increases in the conduction velocity (and consequent reductions in the conduction times) were obtained in both the K and MBS models (Table 1). However, the CL did not appear to be affected by the SEP width, contrary to the findings of the 3D model by Amsaleg et al. (2022). This indeed showed that enlarging the SEPs resulted in loss of driving due to excessive hyperpolarisation arising from a larger sink. This observation was in agreement with a previous study that reported a CL prolongation up to SAN suppression with wider SEPs, while narrower SEPs allowed pacing and driving over a larger range of conductivities (Zyantekorov et al., 2018). This discrepancy may be ascribed to different aspects. First, in a 2D setting like the one considered in this work, the interface between the SAN and crista terminalis grows linearly with increasing SEP width but quadratically in 3D. Second, Zyantekorov et al. (2018) considered SEPs that were open on both sides of the SAN. Third, the K model has a membrane capacitance of 50 pF, which is less than the those used in Amsaleg et al. (2022) (100 pF) and Zyantekorov et al. (2018) (81 pF). All these factors could lower the sink effect in the present model, limiting CL modulation by the SEP width.

### 4.8 Mosaic configuration helps to achieve atrial driving

In the last set of simulations, the MBS model was less easily excitable than the K model; the reason for this is that the present work only involved the 29 equations describing the APs of the atrial cells, while neglecting the ones devoted to modelling the mechanoelectric feedback and calmodulin-kinase activity. Although the AP features were still in accordance with experimental values (Supplementary Table S1), this set of equations was not optimised to describe the electrophysiology of single atrial cells. For example, the maximal upstroke velocity was lower than in the original MBS model ( $dV/dt_{\max}$ = 177 vs 199 mV ms^−1^). Thus, even when the maximal conductance of the fast sodium current was doubled to reproduce the experimental values of the conduction velocity in the crista terminalis, the atrial tissue comprising the MBS model cells struggled to depolarise.

Although further refinements are needed to use the electrophysiological equations of the MBS model in a standalone manner, these simulations provide some useful information on atrial driving. First, atrial cells interspersed in the exit pathways (so-called mosaic model) actually help propagation compared to a flat interface. Although this setup was not needed with the K model, its addition to the gradients in the gap-junctional coupling could represent a more robust and hence more likely anatomical architecture of the SAN (Karpaev et al., 2018; Amsaleg et al., 2022). Therefore, it was included in the full model. Second if the SAN heterogeneity increases the amount of charge delivered to the atrial cells (as discussed above), this is insufficient to overcome the gaps that can be addressed only by coupling or anatomical aspects (such as the gradient and mosaic configurations). Indeed, without the addition of the mosaic setup, only one SAN heterogeneous distribution was shown to drive atrial tissues comprising the MBS model (and to an unphysiologically low rate owing to exit blocks).

### 4.9 Limitations

The present model has several shortcomings. First, to the best of our knowledge, no SAN-specific fibroblast model exists for humans or any other species. In this work, we chose to use the model by Morgan et al. (2016) as it is a more similar phenotype (atrial) to other human fibroblast models (Sachse et al., 2008; Maleckar et al., 2009; Liu et al., 2021).

Second, the fibroblasts were distributed diffusely according to a concentration gradient, with higher amounts distally from the crista terminalis and lower amounts when approaching the ends of the SEPs (Li et al., 2023). However, histological sections of the SANs of different species of mammals showed that the fibroblasts actually formed structural networks supporting and protecting different clusters of SAN myocytes (Camelliti et al., 2004; Sánchez-Quintana et al., 2002; Smithers et al., 2021).

Third, the 2D geometry adopted herein represents a rough simplification of the complex structure of the SAN (Chandler et al., 2011; Fedorov et al., 2010; Csepe et al., 2016) and is simpler than other recent models that also consider fibre direction (Amsaleg et al., 2022; Zhao et al., 2023). Considering that the anatomy has a clear impact on function in an elaborate structure like the SAN (Nikolaidou et al., 2012), our stylised model geometry affects the results quantitatively. For example, conically shaped SEPs may increase the driving strength and limit re-entry occurrence by providing less favourable source–sink balance for the excitations entering the SAN. Moreover, we did not consider the presence of spatially dependent distributions of ACh receptors (Lang et al., 2011) or localisation of different intrinsic pacemaking properties in the specialised SAN clusters (Brennan et al., 2020; Maltsev et al., 2023), which were aspects proposed to explain the shifts in the leading pacemaker location following autonomic modulation.

However, the main drawback of the proposed model is the slow conduction within the exit pathways. Considering a SAN CL of 67 μm, the average conduction velocity in the SAN was ∼ 1 cm s^−1^ (lower than the experimental value of 5 cm s^−1^ reported by Fedorov et al. (2010)), which dropped to < 0.5 cm s^−1^ in the SEPs. These velocities correspond to conduction times exceeding 300 ms in the SEPs (Table 1). Therefore, on average, atrial activation was achieved more than 500 ms after the stimulus originated in the SAN. this value is much higher than in the other models ( < 100 ms reported by Amsaleg et al. (2022) and Li et al. (2020)) as well as outside the experimental range (82 ± 17 ms as reported by Fedorov et al. (2010)). The slow sequence of activation sets the stage for re-entrant activity, as in the H configuration under the control conditions and in the H and HF cases after external pacing (Supplementary Movies S1, S2). Amsaleg et al. (2022) reported velocities as low as 1 cm s^−1^ at the beginning of the gap-junctional gradient in the SEP, but these sharply increased along the SEP. When we used the mosaic architecture with the MBS model, the limited width of the 2D SEP did not allow enough cells for smooth transition to an atrial cell phenotype; thus, the CV remained very low for most of the SEP length before reaching 100 cm s^−1^ just before exiting the SEP.

Finally, as a first approach, all the conditions tested in this work were only with regard to the SAN. This is reasonable considering the presence of fibroblasts and vagal tone, which are known to be minimal in the right atrium under physiological conditions. However, this approach neglects the heterogeneity in the ionic properties of the atrial tissue and its responses to sympathetic stimulation, which likely play roles in modulating the ability of the SAN to drive the atrium.

## 5 Conclusion

Despite the limitations noted above, our simulations predict that heterogeneity and fibroblasts, and particularly their synergistic actions, are beneficial to the physiology of the SAN since they enlarge the parametric space in which the SAN is able to effectively drive the atrium in the 2D human computational models. This represents a fascinating concept since these properties are usually deemed to be detrimental when dealing with the working myocardium. For example, heterogeneity in ventricular repolarisation times has been recognised as a mechanism of onset of arrhythmia and an underlying cause of sudden cardiac death (Amoni et al., 2023). Similarly, both fibroblast proliferation and fibrosis have been shown to serve as substrates for both ventricular and atrial arrhythmias (Nguyen et al., 2014; Pellman et al., 2016; Morgan et al., 2016; Cochet et al., 2018) as well as thought to have roles in sinus node dysfunction (Hao et al., 2011; Wolf et al., 2013; Glukhov et al., 2013; Lou et al., 2014; Glukhov et al., 2015; Kharche et al., 2017; Luo et al., 2013).

One of the reasons why previous computational works have obtained hampered SAN functions when simulating the presence of non-myocardial tissue is that resistive barriers (fibrosis) or passive loads were used as the modelling approaches (Oren and Clancy, 2010; Kharche et al., 2017), as opposed to the presence of fibroblast–myocyte coupling that was evaluated in this work. This overlooks the ability of the fibroblasts to act as current sources when the membrane voltages of the SAN cells are below their resting potentials—a feature that is actually beneficial to their pacemaking activity (Karpaev et al., 2018)—as reported in ventricular tissue cultures (Miragoli et al., 2007).

If validated through experimental studies, these model predictions can provide explanations to the extraordinary amounts of cellular heterogeneity and fibroblasts seen in sinoatrial tissue, without ruling out other hypothesis such as the role of fibroblasts in mechanical protection.

## Data Availability

The code used for this study (simulation and analysis) can be found in: https://github.com/Eugenio95/2D_SAN_ATRIUM. Further inquiries can be directed to the corresponding author.
