# Supplementary material for: Sinoatrial node heterogeneity and fibroblasts increase atrial driving capability in a two-dimensional human computational model
Source: Front Physiol. 2024 Jul 30;15:1408626. doi: 10.3389/fphys.2024.1408626 (PMC11319284; doi:10.3389/fphys.2024.1408626)
Supplement: Supplementary file 3 [file Presentation1.pdf]

Supplementary Material

1 SUPPLEMENTARY DATA

In order to allow comparisons with clinical-level and experimental data, simulated electrograms (EGMs) were computed as previously done in 2D in our group Bartolucci et al. (2021) according to Shillieto et al. Shillieto et al. (2016). A 7-french bipolar sensing catheter with $3.5\,\mathrm{mm}$ tip and 2-5-2 mm inter-electrode distances, as used in Li et al. (2017), was implemented. Considering a $100\,\mu\mathrm{m}$ cell length for the Koivumäki model, this converted to 20-50-20 cell distances in the discrete model (Figure SS1). Activation time maps were built by color-coding the time difference in the occurrence of the peak of the membrane voltage first derivative. The atrial cells at the SEP frontiers which first showed an AP were taken as zero-reference.

In order to simulate simultaneous administration of ACh and ISO, the FWS model Fabbri et al. (2017) was modified to take into account their effects in an additive manner. In the model, the only target shared by parasympathetic and sympathetic stimulation is the parameter b_{up}, the basal uptake rate of the SERCA pump. Its dependence on ACh and ISO concentrations was thus modified according to the following equation:

$$b_{up} = \frac{0.7 * [ACh]}{0.0009 + [ACh]} - 0.25$$

where the first term represents the dose-dependent ACh response and the second one a fixed reduction due to ISO at a fixed concentration of $1\,\mu\mathrm{M}$.

2 SUPPLEMENTARY FIGURES, TABLES AND MOVIES

2.1 Supplementary Figures

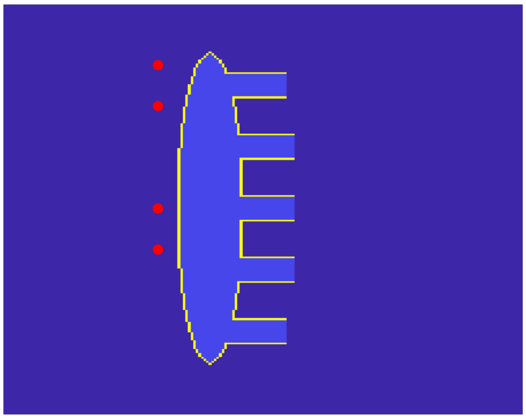

Figure S1. EGM catheter position inside the tissue model. The red dots indicate the single electrode positions with the 2-5-2 mm spacing, numbered #1-4 from top to bottom.

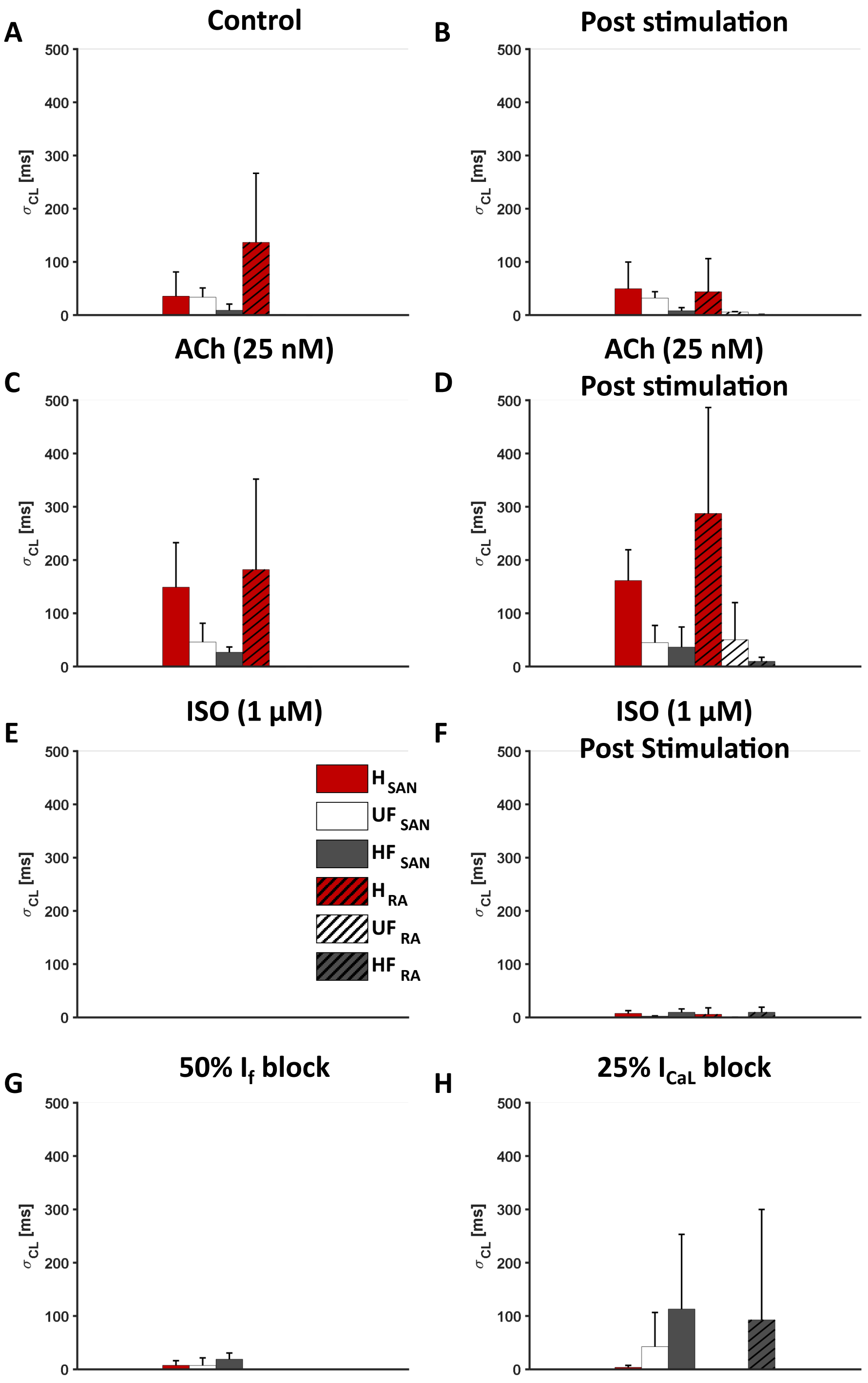

Figure S2. SAN and RA CL standard deviation of the human model in different conditions. In the SAN, σ_{CL} reports SAN myocytes synchronization; in the RA (hatched bars), it can inform on the presence of SEP alternans or exit blocks. H: heterogeneity; UF: fibroblasts; HF: heterogeneity and fibroblasts.

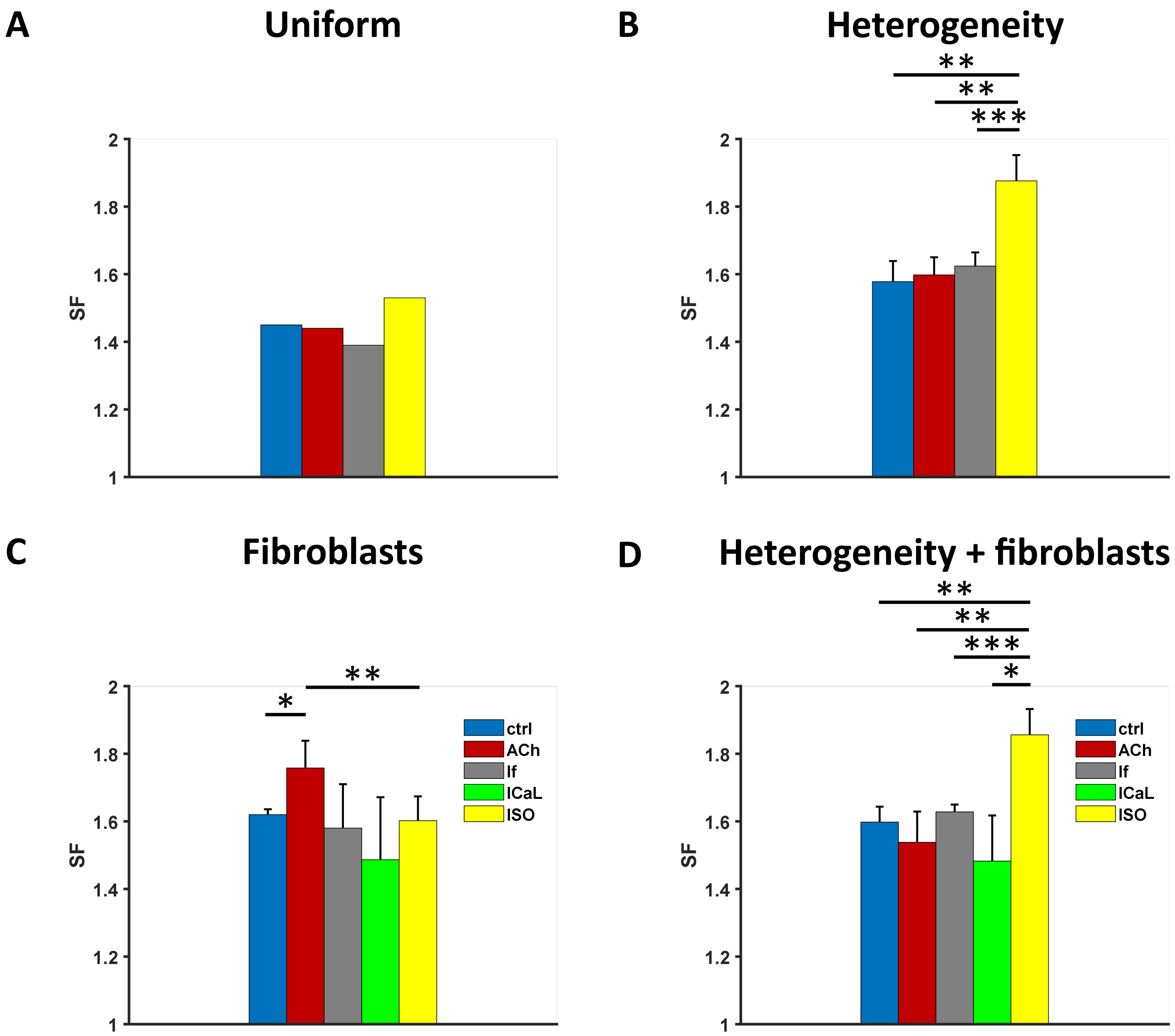

Figure S3. Comparisons of safety factor values across the different human model set-ups in different conditions. A) Uniform set-up. B) Presence of heterogeneity. C) Presence of fibroblasts. D) Presence of heterogeneity and fibroblasts. No atrial driving was achieved with 25% I_{CaL} block in A) and B).

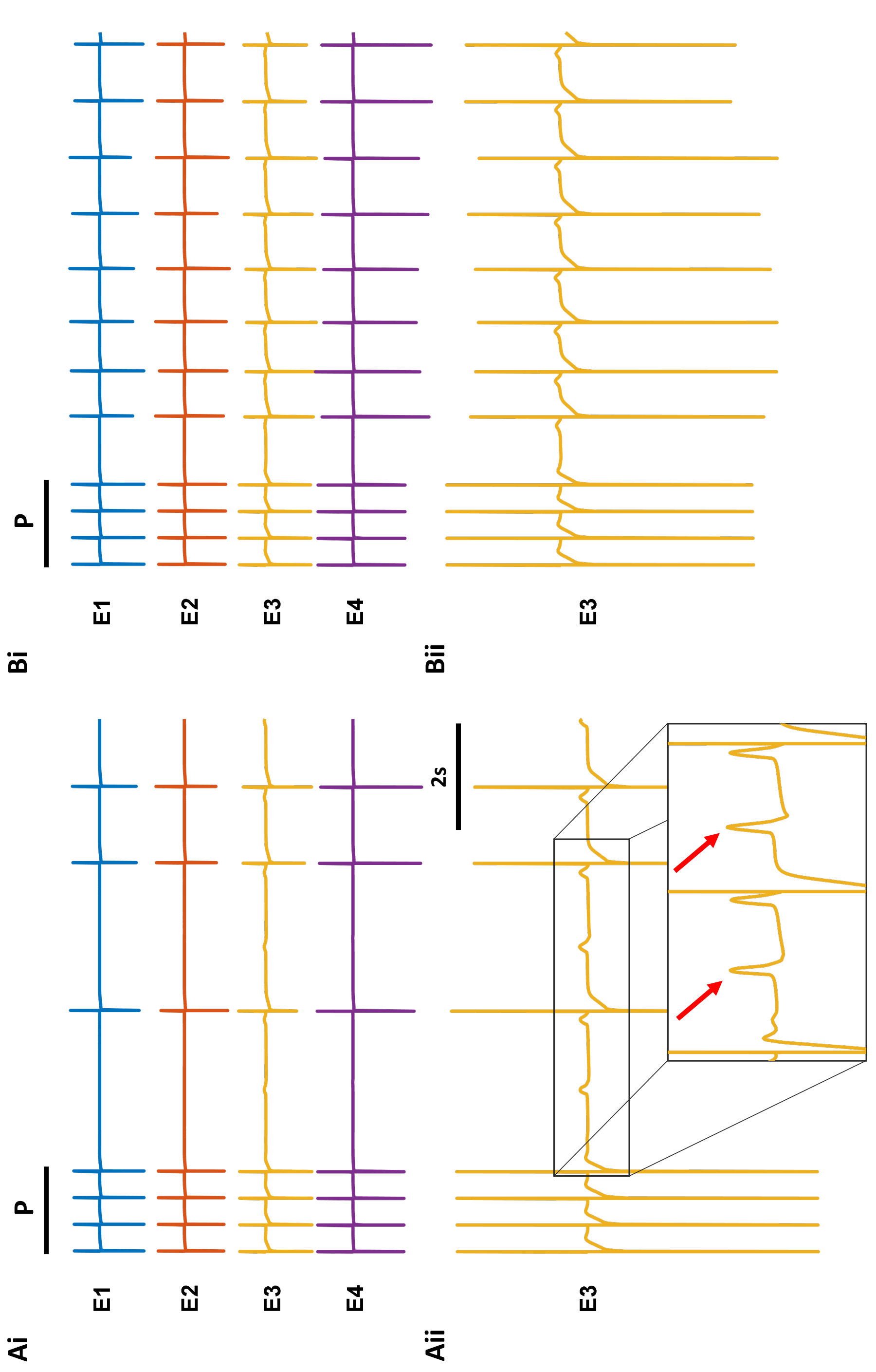

Figure S4. Electrograms in the tissue with ACh administration during and after pacing. Ai) Uniform SAN tissue recordings with the 4 electrodes of Figure SS1; Aii) Electrode #3 signal with zoom on the activity after pacing is stopped. The red arrow indicates SAN activity not followed by atrial excitation (exit blocks). Bi) HF tissue #1 recordings and Bii) zoom on electrode #3 showing no exit blocks. P: pacing; E1-4: electrodes 1-4.

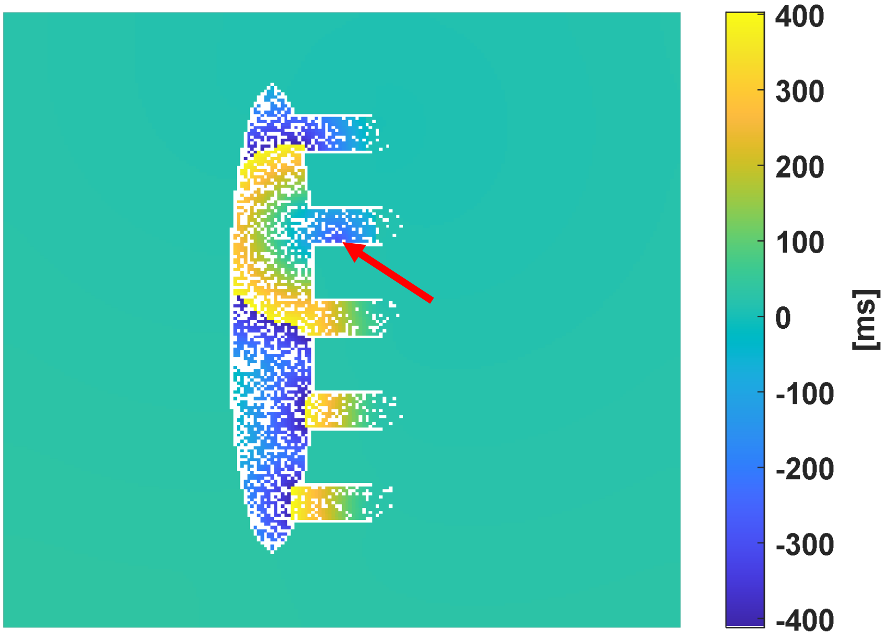

Figure S5. Activation time map of tissue #2 of the HF set-up with 25% I_{CaL} block. Atrial activation at the SEP interface is taken as the reference instant. As pointed by the red arrow, activation starts in SEP #2 and travels both retrograde inside the SAN and forward to excite the atrium. White pixels represent fibroblasts.

2.2 Supplementary Tables

Table S1. Biomarkers of the reduced MBS model. Experimental data is reported as mean and range [lower bound, upper bound]. APD: action potential duration at 90, 50 and 30% of repolarization; V_{amp}: voltage difference between overshoot and resting membrane potential; V_{diast}: resting membrane potential; dV/dt_{max}: maximal upstroke velocity; Ca_{sys}: systolic calcium concentration in the cytosol; Ca_{dias}: diastolic calcium concentration in the cytosol; ttp_{ca}: time to calcium concentration peak in the cytosol; rt_{50Ca}: rise time to 50% calcium concentration peak in the cytosol; TT_{Ca}: total transient duration. Definitions as in Mazhar et al. (2023).

Biomarker	Experimental data	Original MBS	Reduced MBS
**Action potential**			
$\mathbf{APD_{90}}$ [ms]	263 [202, 332]	225	238
$\mathbf{APD_{50}}$ [ms]	50 [25, 94]	45	50
$\mathbf{APD_{30}}$ [ms]	8 [5, 14]	8	14
$\mathbf{V_{amp}}$ [mV]	103 [83 130]	120	112
$\mathbf{V_{rest}}$ [mV]	-75 [-68, -75]	-75	-76
$\mathbf{dV/dt_{max}}$ [mV ms^{-1}]	179 [159, 232]	199	177
$\mathbf{Ca^{2+}}$ **transient**			
$\mathbf{Ca_{sys}}$ [µM]	0.268 [0.18, 0.4]	0.221	0.519
$\mathbf{Ca_{dias}}$ [µM]	0.223 [0.2, 0.25]	0.187	0.172
$\mathbf{ttp_{Ca}}$ [ms]	53 [49, 56]	94	53
$\mathbf{rt_{50Ca}}$ [ms]	178 [169, 187]	173	106
$\mathbf{TT_{Ca}}$ [ms]	539 [508, 570]	690	404

Table S2. Action potential features in the SAN in different set-ups in basal conditions. The H, UF and HF set-up reported are for tissue #1.

Feature	U	H	UF	HF
**Whole SAN**				
**CL [ms]**	810±52.5	624±9.3	815±32.5	639±0.7
**MDP [mV]**	-58.5±1.6	-56±2.5	-55.8±2.6	-54.4±6.4
$\mathbf{dV/dt_{max}}$ **[mV ms^{-1}]**	7.4±1.3	8.5±1.9	7.9±2.2	8.4±2.6
**Leading SEP only**				
**CL [ms]**	814±0.1	624±1.9	817±0.1	639±0.1
**MDP [mV]**	-55.7±2.2	-56.1±2.4	-57.8±5.3	-59±6.4
$\mathbf{dV/dt_{max}}$ **[mV ms^{-1}]**	7.7±4.4	7.6±3.7	9.3±5.6	10.4+6.9

Table S3. Action potential features in the SAN with different set-ups with 25% I_{CaL} block. The H, UF and HF set-up reported are for tissue #1.

Feature	U	H	UF	HF
**Whole SAN**				
**CL [ms]**	1131±0.1	884±1.9	1073±155	805±24.9
**MDP [mV]**	-58.5±2.1	-55.7±2.5	-54.9±1.9	-53.6±3
dV/dt_{max} **[mV ms^{-1}]**	5.4±0.6	6.1±1.2	5.5±0.7	6.5±2.4
**Leading SEP only**				
**CL [ms]**	1131±0.1	883±4.1	1296±433	806±0.1
**MDP [mV]**	-59.1±5.2	-58.9±5.2	-55.8±3.9	-55.1±3.4
dV/dt_{max} **[mV ms^{-1}]**	5.4±1.5	5.8±1.7	5.1±1.8	7.2±4.6

2.3 Supplementary Movies

Movie S1. Electrical activity after external 2 Hz pacing in the H tissue #2.

Movie S2. Electrical activity after external 2 Hz pacing in the HF tissue #1.

Movie S3. Electrical activity after external 2 Hz pacing in the U model with 25 nM ACh.

Movie S4. Electrical activity in the HF tissue #3 with 25% I_{CaL} block.

Movie S5. Electrical activity in the full model with $R_{gap} = 100$ MΩ for the fibroblasts.

Movie S6. Electrical activity in the full model with $R_{gap} = 1$ GΩ for the fibroblasts.

REFERENCES

Bartolucci, C., Fabbri, C., Tomasi, C., Sabbatani, P., Severi, S., and Corsi, C. (2021). Computational Analysis of Mapping Catheter Geometry and Contact Quality Effects on Rotor Detection in Atrial Fibrillation. *Frontiers in Physiology* 12. doi:10.3389/fphys.2021.732161

Fabbri, A., Fantini, M., Wilders, R., and Severi, S. (2017). Computational analysis of the human sinus node action potential: model development and effects of mutations: Model of the human sinoatrial AP. *The Journal of Physiology* 595, 2365–2396. doi:10.1113/JP273259

Li, N., Hansen, B. J., Csepe, T. A., Zhao, J., Ignozzi, A. J., Sul, L. V., et al. (2017). Redundant and diverse intranodal pacemakers and conduction pathways protect the human sinoatrial node from failure. *Science Translational Medicine* 9, eaam5607. doi:10.1126/scitranslmed.aam5607

Mazhar, F., Bartolucci, C., Regazzoni, F., Paci, M., Dedè, L., Quarteroni, A., et al. (2023). A detailed mathematical model of the human atrial cardiomyocyte: integration of electrophysiology and cardiomechanics. *The Journal of Physiology* n/a. doi:10.1113/JP283974

Shillieto, K. E., Ganesan, P., Salmin, A. J., Cherry, E. M., Pertsov, A. M., and Ghoraani, B. (2016). Catheter simulator software tool to generate electrograms of any multi-polar diagnostic catheter from 3D atrial tissue. In *2016 38th Annual International Conference of the IEEE Engineering in Medicine and Biology Society (EMBC)*. 2741–2744. doi:10.1109/EMBC.2016.7591297
